# Prevalence and associated factors of metabolic syndrome in adults: a population-based epidemiological survey in Jiangxi province, China

**DOI:** 10.1186/s12889-020-8207-x

**Published:** 2020-01-30

**Authors:** Li Ting Wu, Yun Feng Shen, Lei Hu, Mei Ying Zhang, Xiao Yang Lai

**Affiliations:** 1 Department of Endocrinology and Metabolism, The Second Affiliated Hospital of Nanchang University, No.1, Minde Road, Donghu District, Nanchang, 330006 China; 2 Department of Endocrinology and Metabolism, The First Affiliated Hospital of Sun Yat-sen University, No. 58, ZhongshanEr Road, Guangzhou, 510080 China; 3 Institute for the Study of Endocrinology and Metabolism Diseases in Jiangxi Province, No.1, Minde Road, Donghu District, Nanchang, 330006 China

**Keywords:** Metabolic syndrome, Prevalence, Associated factors

## Abstract

**Background:**

Metabolic syndrome (MS) has abruptly increased in China in the past two decades, gradually representing an important public health threat over the years. Here, we firstly reported the prevalence and associated factors of metabolic syndrome in Jiangxi province, China.

**Methods:**

A population-based cross-sectional survey was performed in Jiangxi province, China, from April to August 2015. MS was diagnosed by International Diabetes Federation (IDF) and Chinese Diabetes Society (CDS) criteria, respectively. Factors associated with MS were investigated by multivariate logistic regression.

**Results:**

A total of 2665 residents aged over 18 years were enrolled, and 2580 effectively participated. According to IDF and CDS criteria, age-standardized prevalence of MS were 21.1 and 15.2% in all participants, respectively; prevalence were 19.6% or 17.1% in men, and 22.7% or 13.0% in women, based on these respective criteria. Rural participants had a significantly higher prevalence than urban individuals, so did rural females. Prevalence in males did not differ between rural and urban participants. Factors independently associated with MS were low education level and menopausal state.

**Conclusions:**

To the best of our knowledge, this was the latest study on MS prevalence in Jiangxi province. In conclusion, MS prevalence is high in Jiangxi province. Considering the unhealthy lifestyle, education is urgently needed to prevent the rapid increase of MS prevalence.

## Background

Metabolic syndrome (MS) is a constellation of interrelated metabolic disturbances based on insulin resistance, including visceral adiposity, atherogenic dyslipidemia, elevations of blood pressure (BP) and glucose, endothelial dysfunction, prothrombotic and proinflammatory states; it is associated with greatly increased morbidity and mortality of cardiovascular disease (CVD) and type 2 diabetes mellitus (T2DM) [1–5]. In the global context of urbanization and the spread of unhealthy life-style, the high prevalence of MS gradually over-burdens public health [6].

Interestingly, different MS prevalence have been presented in published studies assessing the Chinese population [7–17]. MS prevalence was reported at 13.3% in 2001, rapidly rising to 18.2% in 2009 in China Health and Nutrition Survey, according to the Chinese Diabetes Society (CDS) criteria [7, 14]. Paradoxically, a prevalence of 9.82% found in 2014 was lower than that of 2009 by the same criteria [15]. To the best of our knowledge, at least three reasons may explain these results. Firstly, diverse dietary habits in different regions could represent an important cause of the disparate prevalence of MS. The MS prevalence in Jilin province and the Yan-an region of Shanxi province were 22.4 and 26.4% according to International Diabetes Federation (IDF) criteria, respectively [13, 18]. Secondly, different diagnosis criteria also could contribute to these differences. As shown previously, MS prevalence was 12.6% by the CDS criteria in the Hangzhou region of Zhejiang province, and 7.3% by IDF criteria in Guangdong province [11, 12]. Thirdly, different socioeconomic status (SES) might be a vital factor leading to the differences in MS prevalence. As reported previously, MS prevalence was 33.9% by modified IDF criteria in affluent urban districts in Shanghai, and 11.8% by IDF criteria in un-developed rural regions of NingXia province [16, 17].

Both IDF and CDS criteria for MS are two widely used criteria for clinical and research purposes [9, 14]. Most components of MS are similar between IDF and CDS criteria, although some slight differences remain. IDF criteria emphasize on central obesity evaluated by waist circumstance (WC), while CDS criteria stressed on overall body obesity evaluated by body mass index (BMI). Besides, IDF criteria consider central obesity as prerequisite, and have stricter cut-offs for blood pressure and blood glucose levels.

Jiangxi province is an under-developed province in central China, with 45 million Han Chinese or so. With sustained economic growth, the Western lifestyle in local populations has been developed in the past two decades. Unfortunately, although many studies of MS have been performed in other regions of China, little information is available for Jiangxi province. In the present study, we investigated MS prevalence based on the IDF and CDS criteria in Jiangxi province, in a cross-sectional survey sponsored by the TIDE (Thyroid disorders, Iodine status and Diabetes: a national Epidemiological survey) study group.

In terms of the associated factors of MS, education level, gender, residential region and income are proposed to be related with MS [19]. But these factors may be correlated with different SES or criteria used for defining MS [20]. Moreover, they might have region-specificity [21]. Therefore, the current study subsequently determined the independently associated factors for MS in Jiangxi province.

## Methods

### Study design and population

The current data originated from the epidemiological data collected by the TIDE. This population-based, cross-sectional, epidemiological survey was performed in Jiangxi province from April to August in 2015. Sample size was estimated to meet generally recommended requirements for precision in a random sampling survey. Assuming the confidence interval was 95% and the MS prevalence 10%. The permissible relative sampling error was 4%. Therefore, the total sample size was 217. In this study, 2665 adults were invited and 2580 effectively attended, giving a response rate of 96.8%. The sample size achieved the minimum requirement for the sampling method.

**Fig. 1:**
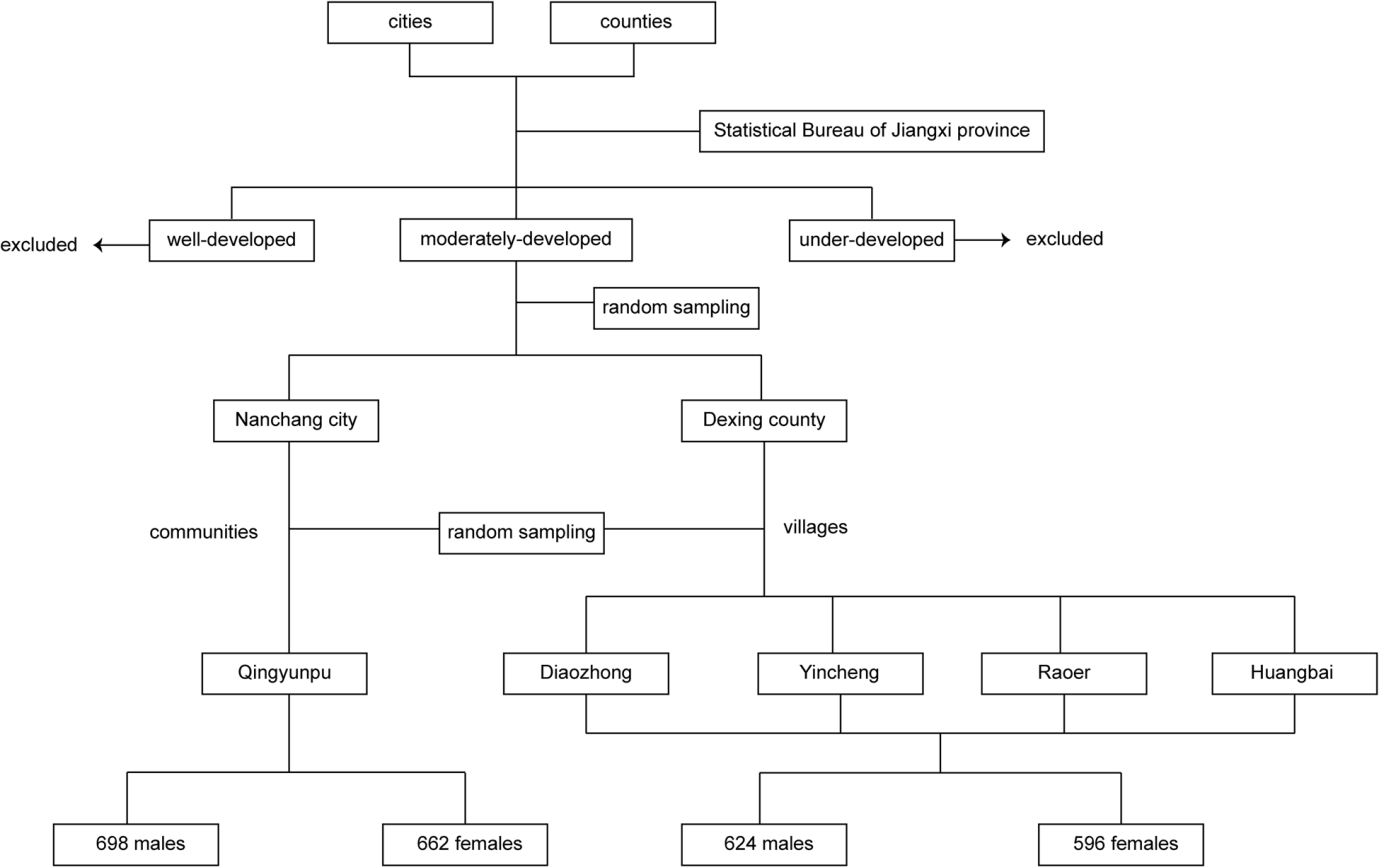
Flow chart of the cluster sampling process

A cluster sampling method was applied in this study (Fig. 1). (1) According to the economical size of each city and county in Jiangxi province provided by Statistical Bureau, areas which fell in the 25th to 75th percentiles of the Gross Domestic Product (GDP) level were included. Besides, considering the data accessibility, sparsely inhabited districts with higher or lower economical sizes were excluded. (2) In the above areas, one urban city (Nanchang) and one rural county (Dexing) were chosen using a simple random sampling method. Afterwards, one community (Qingyunpu) and four villages (Diaozhong, Yincheng, Raoer and Huangbai) were picked using the above method. (3) The participants were restricted to local residents aged over 18 years old who had been living at the study sites for at least 5 years. Pregnant women were excluded. According to residential registration, all of the eligible candidates were included.

### Questionnaire, anthropometric measurements and blood sample investigation

Firstly, individual demographic characteristics and disease history questionnaire were completed. Demographic and clinical information included age, sex, level of education, marital status, menstrual condition, family annual income, occupational status, smoking, history of diseases such as diabetes, hypertension, dyslipidemia and hyperuricemia, medications for diseases and family history of diabetes. In multiple logistic regression analysis, age was classified into 3 groups, including ≤40, 41–65 and ≥ 66 years old. Education was classified into 3 levels, including primary school and below, middle and high school, and college and above. Family annual income was graded into 4 levels: (1) < 30,000; (2) 30,000~50,000; (3) 50,000~100,000; (4) > 100,000 Chinese yuan (100 CNY = 14.56USD). Occupational status was categorized into student, worker (farmer), house worker (retired), clerk and others. Smoking was defined as at least 100 cigarettes consumed prior to the survey and was further divided into two groups on the basis of the frequency of cigarettes consumption (< 1 cigarette per day or ≥ 1 cigarette per day). Amenorrhea was defined as not having a period for at least 6 months or 3 menstrual cycles.

Anthropometric data, including BP, heart rate (HR), WC, height and body weight, were measured by recommended standard procedures. In brief, BP and HR were average values of two separate measurements taken at 5-min intervals. Weight and height were measured without shoes or heavy garments. BMI (kg/m^2^) was determined by dividing the weight (kg) by height (m) squared. WC was measured in the erect position at the middle of the lowest rib and the superior border of the iliac crest.

Blood samples were obtained after at least 10 h of fasting to determine fasting blood chemistry parameters. Afterward, all subjects were given a standard 2 h-75 g oral glucose tolerance test (OGTT). All serum parameters were detected on a Mindray (Mindray Medical International Limited, China) automatic biochemistry analyzer. Serum total cholesterol (TC) and triglyceride (TG) levels were determined by enzymatic methods. Serum low density lipoprotein cholesterol (LDL-C) and high density lipoprotein cholesterol (HDL-C) amounts were measured by the direct method. Serum uric acid (UA) content was measured by the uric acid enzyme-peroxide enzyme coupling method. Fasting plasma glucose (FPG) and OGTT-2 h plasma glucose (2 h PG) were determined by the glucose oxidase method. Glycosylated hemoglobin A1c (HbA1c) was measured by high pressure liquid chromatography. All the procedures were executed by experienced laboratory technicians.

### Diagnosis of metabolic syndrome

MS was diagnosed based on IDF or CDS criteria (Chinese specific) [9, 14]. These two criteria are described in Additional file 1: Table S1.

### Data collection and statistical analyses

In this study, data collection was performed by trained medical professionals at the Second Affiliated Hospital of Nanchang University. Data analysis was limited to individuals who had completed all procedures, comprising 1322 (51.2%) males and 1258 (48.8%) females; 1360 (52.7%) participants lived in urban areas, and 1220 (47.3%) in rural regions.

An EpiData (EpiData Association, Odese, Denmark) database was established, and all data were analyzed by SPSS (Statistical Program for Social Sciences, version 20.0). Continuous variables were described as mean (standard error, SE) and analyzed by student’s *t*-test. Categorical variables are presented as number and percentage, and analyzed by the Chi-square test. The official 2010 census data of China was used to determine age-standardized ratios.

We analysis the associated factors for MS with logistic regression models. We reported the odd ration (OR) with 95% CI. Covariates included in the multivariable logistic regression models were profession (student [reference] vs worker vs clerk vs houseworker vs others), family annual income (below 30,000 [reference] vs 30,000–50,000 vs 50,000–100,000 vs above 100,000), age (18–40 [reference] vs 41–65 vs above 66), education level (primary school or below [reference] vs middle or high school vs college or above), family history of diabetes (yes [reference] vs no), smoke (no or less than 1 cigarette/d [reference] vs more than 1 cigarette/d), Menses condition (menopausal state [reference] vs un-menopausal state). We also investigated whether the associated factors for MS varied by region and gender by stratified multivariable analyses (in urban males, urban females, rural males and rural females respectively).

## Results

### Characteristics of the survey population

**Fig. 2:**
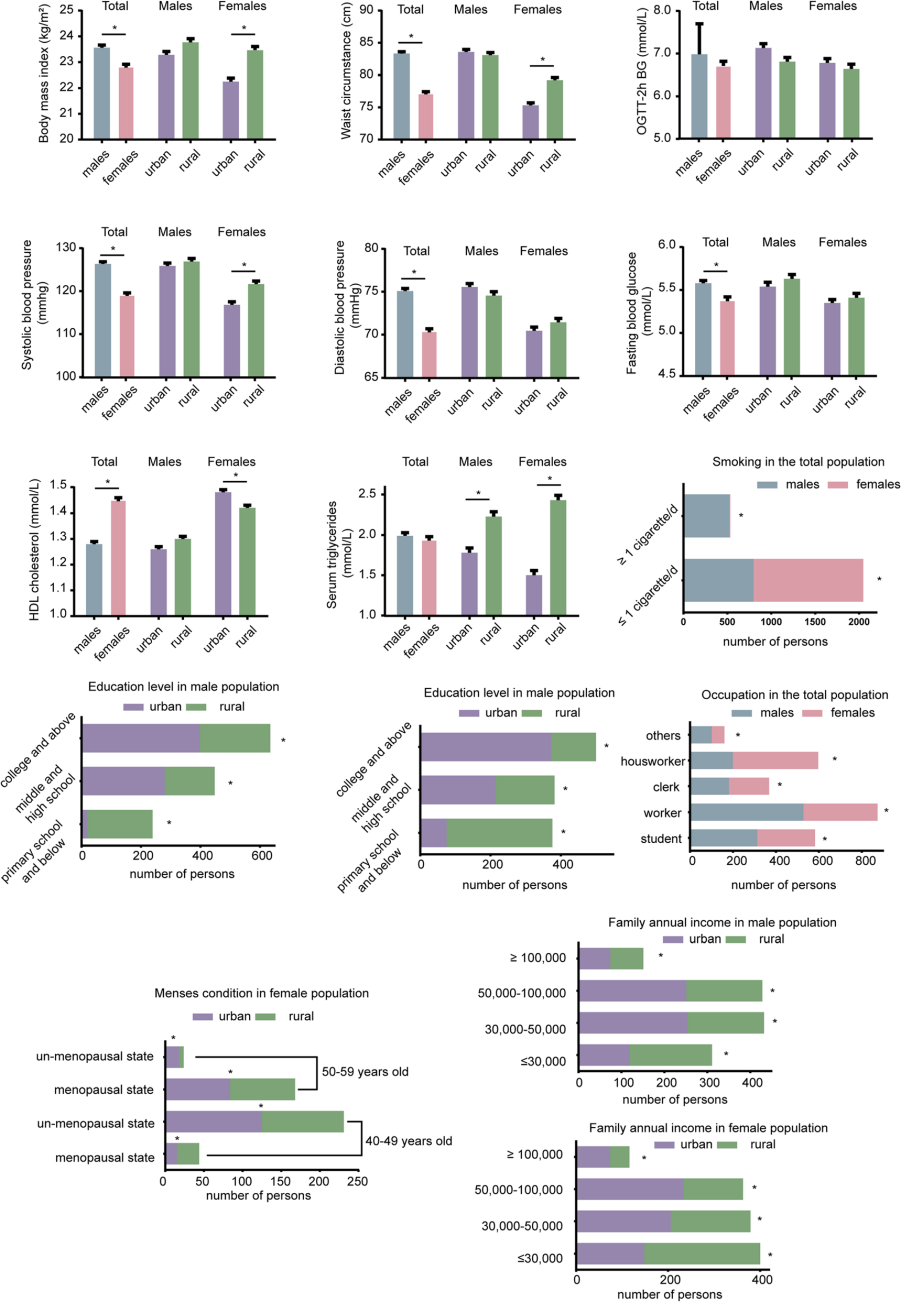
Characteristics of the survey population. * *p* < 0.05 for men vs women; or urban vs rural as indicated in the figure. HDL cholesterol, high density lipoprotein cholesterol; OGTT-2 h BG, blood glucose of 2 h oral glucose tolerance test

A total of 1322 adult males and 1258 adult females were finally included and evaluated in this cross-sectional study. The characteristics of these individuals are shown in Fig. 2. Though no significant differences were found in TG levels between men and women, rural participants showed significantly higher levels than urban counterparts. Men had higher BMI, WC, SBP and DBP, but HDL-C levels were higher in women. No significant differences were seen in BMI, WC and SBP between urban and rural men. In contrast, rural women had greater levels of the above anthropometric values than urban women. For HDL-C, rural women showed significantly lower levels. Besides, urban women had delayed menopause compared with their rural counterparts. In urban areas, participants had higher education and income levels. The smoking rate was nearly 40% in men and was significantly higher than that of women. Meanwhile, more women performed housework than men.

### MS prevalence based on different criteria

**Fig. 3:**
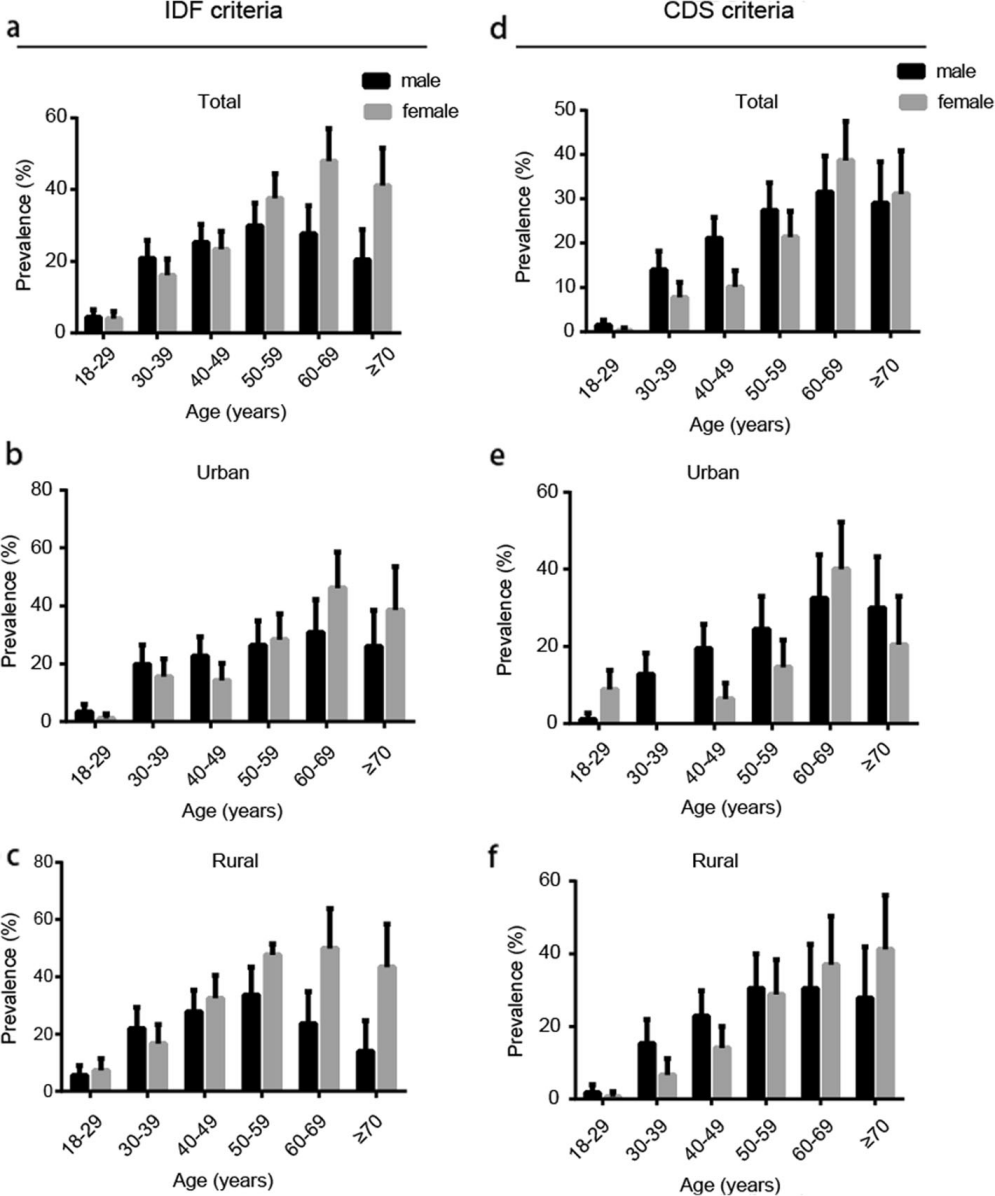
Age-specific and age-standardized prevalence of MS in Jiangxi Province in 2015, based on IDF (**a**-**c**) and CDS (**d**-**f**) criteria, respectively. Error bars indicate 95% confidence intervals

**Fig. 4:**
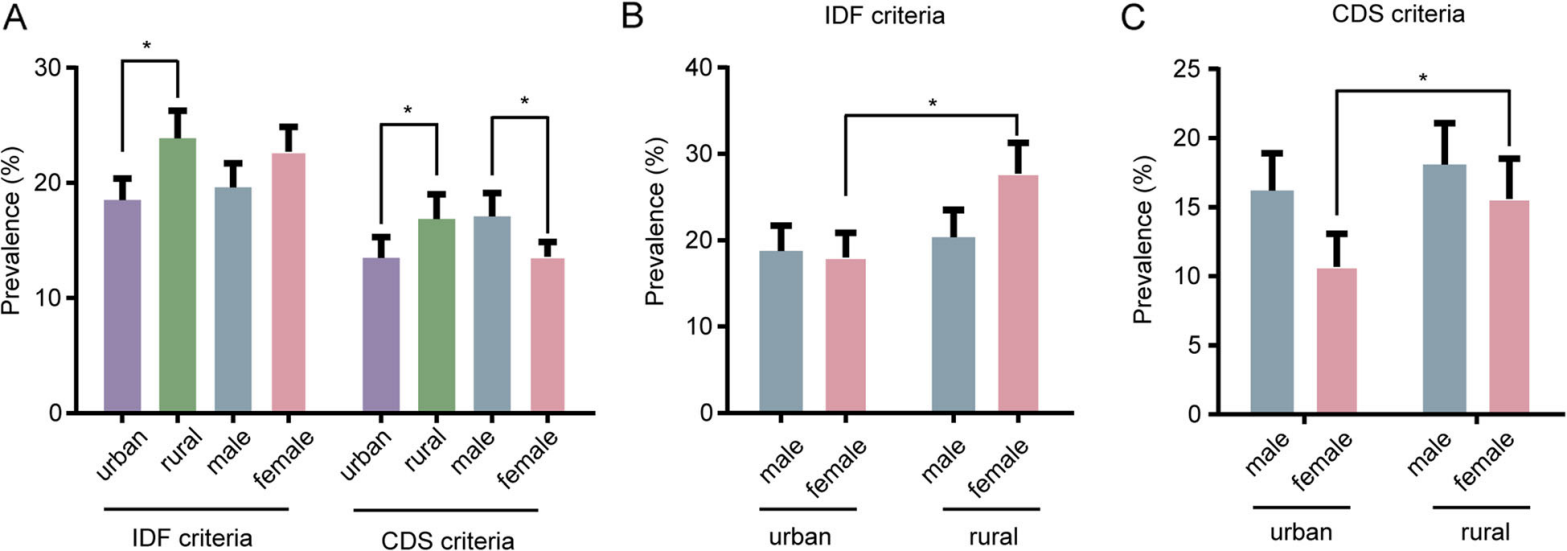
Region- and sex-specific prevalence of MS in Jiangxi Province in 2015, based on IDF (**a** and **b**) or CDS (**a** and **c**) criteria. Error bars indicate 95% confidence intervals. * *p* < 0.05 as indicated in the figure

The prevalence of MS are displayed in Table 1. Of the 2580 subjects, 542 (21.0%) were diagnosed with MS based on IDF criteria. The age-adjusted prevalence was 21.1% for the whole population, with 22.6 and 19.6% in females and males, respectively. Significant differences were observed between rural (23.9%) and urban (18.5%) areas, especially in female population (Fig. 4). With population aging, MS prevalence increased (Fig. 3). No significant gender differences were observed in total prevalence, but women had higher prevalence in elderly groups than men (47.9% vs 27.6 and 41.1% vs 20.4%, in 60–69 and > 70 years, respectively, *p* < 0.01). In peri-menopausal women groups, MS prevalence in rural areas were significantly higher compared with those of urban regions (32.6% vs 14.3 and 47.8% vs 28.4%, in 40–49 and 50–59 years, respectively, *p* < 0.01). Besides, such difference was also found in 18–29 years females (7.3% vs 1.1%, respectively, *p* < 0.01).

**Table 1:** Age- and sex-specific prevalence (%) of MS based on different definitions

	Men	IDF	Total	Men	CDS	Total
		Women			Women	
Urban						
18–29	3.4	1.1 **	2.3 **	1.1	0	0.6
30–39	19.9	15.6	17.8	12.8	8.9	10.9
40–49	22.7	14.3 **	18.7 **	19.5*	6.4 **	13.3
50–59	26.4	28.4 **	27.4 **	24.5	14.7 **	19.7 **
60–69	30.9	46.2	38.4	32.4	40.0	36.1
≥ 70	26.0	38.6	31.9	30.0	20.5	25.5
Total, crude	18.8	18.0 **	18.5 **	16.2 *	10.7 **	13.5 **
Rural						
18–29	5.5	7.3	6.4	1.8	0.7	1.3
30–39	22.0	16.7	19.3	15.4 *	6.7	11.1
40–49	27.9	32.6	30.2	22.9	14.1	18.5
50–59	33.7	47.8	40.5	30.5	28.9	29.7
60–69	23.7 *	50.0	36.3	30.5	37.0	33.6
≥ 70	14.0 *	43.5	29.2	27.9	41.3	34.8
Total, crude	20.4 *	27.7	23.9	18.1	15.6	16.9
total population						
18–29	4.4	4.0	4.2	1.5	0.3	0.9
30–39	20.8	16.1	18.5	14.0 *	7.8	11.0
40–49	25.2	23.3	24.3	21.1 *	10.2	15.8
50–59	29.9	37.5	33.6	27.4	21.4	24.4
60–69	27.6 *	47.9	37.4	31.5	38.7	35.0
≥ 70	20.4*	41.1	30.6	29.0	31.1	30.1
Age-standardized	19.6	22.7	21.1	17.1*	13.0	15.2

* *p* < 0.05 for men vs women; ** *p* < 0.05 for urban vs rural

Of the 2580 subjects, 390 (15.1%) were diagnosed with MS by CDS criteria. The age-adjusted prevalence was 15.2% for the whole population, including 17.1% in men and 13.0% in women. Similarly, there was a significant age-related increase in MS prevalence (Fig. 3). MS prevalence was significantly higher in men compared with women (*p* < 0.01), especially in the 30–39 (14.0% vs 7.8%, *p* < 0.05) and 40–49 (21.1% vs 10.2%, *p* < 0.05) years age groups. In peri-menopausal women, MS prevalence in rural areas were significantly higher than those of urban regions (14.1% vs 6.4 and 28.9% vs 14.7% in the 40–49 and 50–59 years age groups, respectively, *p* < 0.05). Similar to IDF criteria, rural participants showed significantly higher prevalence than urban counterparts. Besides, rural females had significantly higher prevalence (Fig. 4).

### Independent associated factors for MS

Overall, the multivariate adjusted OR for MS in CDS criteria continually decreased with increasing education level, from 0.851 (0.645–1.123) in the middle or high school group to 0.670 (0.512–0.877) in the college and above group. Unfortunately, we haven’t identified any of associated factors for MS by IDF criteria. Hence, we performed further analysis based on CDS criteria. In stratified logistic regression analysis, we found that the risk of developing MS is 69% less in middle or high school group and 76% less in college or above group than primary school or below group (reference) in men from urban regions. Higher education level did not protect women from MS in urban areas. Compared with non-menopausal women, menopausal females had a higher risk of developing MS, by about 1.5-fold for urban females. Occupation, living area, elderly age, family annual income, smoking and family history of T2DM were not independently associated with MS in either gender (Table 2).

**Table 2:** Independent associated factors of MS using multiple logistic regression

Participants		95.0% CI for Exp(B)	
Factors	OR/Exp(B)	Lower	Upper
All participants			
Education			
Primary school or below (reference)	1.0	1.0	
Middle or high school	0.851	0.645	1.123
College or above	0.670	0.512	0.877
Urban males			
Education			
Primary school or below (reference)	1.0	1.0	
Middle or high school	0.310	0.116	0.823
College or above	0.237	0.090	0.624
Urban females			
Menses condition			
menopausal state (reference)	1.0	1.0	
un-menopausal state	0.645	0.422	0.984

*OR* odds ration, *CI* confidence interval

### Prevalence of individual metabolic abnormalities

Using the IDF definition of MS, high TG level was the most frequent element in both genders. The prevalence of high blood pressure was two times higher in men than in women (Table 3). Using CDS definition of MS, high blood glucose level was the most frequent, while high HDL cholesterol level the least (Table 4).

**Table 3:** Sex-specific prevalence (%) of individual metabolic abnormalities based on IDF definition

Metabolic abnormality	Prevalence					
	Men (*N* = 258)		Women (*N* = 164)		Total (*N* = 390)	
	%	95% CI	%	95% CI	%	95% CI
Obesity	34.1	28.3–39.9	37.7	32.0–43.3	34.1	30.1–38.1
High serum TG level	49.6	43.5–55.8	41.5	35.8–47.3	45.4	41.2–49.6
High HDL cholesterol	19.8	14.9–24.7	32.0	26.6–37.5	26.2	22.5–29.9
High blood pressure	45.0	38.9–51.1	22.2	17.3–27.0	33.0	29.1–37.0
High serum glucose level	31.0	25.3–36.7	23.9	19.0–28.9	27.3	23.5–31.1

**Table 4:** Sex-specific prevalence (%) of individual metabolic abnormalities based on CDS definition

Metabolic abnormality	Prevalence					
	Men (*N* = 258)		Women (*N* = 164)		Total (*N* = 390)	
	%	95% CI	%	95% CI	%	95% CI
Obesity	33.6	27.4–39.8	31.7	24.5–38.9	32.8	28.1–37.5
High serum TG level	39.4	33.0–45.8	55.5	47.8–63.2	46.2	41.2–51.1
High HDL cholesterol	7.1	3.7–10.4	6.1	2.4–9.8	6.7	4.2–9.2
High blood pressure	23.0	17.5–28.5	24.4	17.7–31.0	23.6	19.4–27.8
High serum glucose level	42.0	35.6–48.5	67.1	59.8–74.3	52.6	47.6–57.5

## Discussion

In this population-based, cross-sectional, epidemiological survey, we found that overall age-adjusted prevalence of MS were 21.1 and 15.2%, according to IDF and CDS criteria, respectively. MS prevalence gradually increased with age. Rural individuals, especially females, had higher prevalence of MS, irrespective of the diagnostic criteria used (IDF or CDS criteria). However, different diagnostic criteria revealed opposite outcomes regarding sex prevalence. Moreover, low education level and the menopausal state could be independent associated factors for MS.

MS increases the risk of T2DM, CVD and cancer in the general population, resulting in an enormous economic burden for the society [1, 5]. MS prevalence were 19.85 and 9.95% in 2013 in Jiangxi province based on IDF and CDS criteria, respectively, as reported by Cheng et al. [22].. After 2 years, a significant increase was observed in this survey. This terrible phenomenon suggests that effective prevention measures should be taken immediately.

A higher prevalence of MS for the elderly has been observed in worldwide surveys [6, 12, 18, 23, 24]. With increased population aging in China, MS-related economic burden would challenge medical workers and public health. In this study, MS prevalence increased with age and reached a peak in 60–69 years old individuals diagnosed by both criteria. However, according to IDF criteria, the male prevalence rate peaked at 50–59 years old. These trends were also reported by Li et al. [13] in the Yan-an region. In contrast, peak ages were reduced than those of Chen’s study in Shanghai [25]. For preventing morbidity and mortality of MS related diseases, more attention should be focused on individuals of 50–69 years old in Jiangxi province.

A difference in MS prevalence may exist due to diverse living areas from north to south China. Though close crude prevalence were shown between our results and 2013 data in the Chinese population, a higher MS prevalence was reported in northern China, for example in the Yan-an and Jilin regions [10, 13, 26]. Besides, a more rapid economic development compared with the national average in Jiangxi in recent years has contributed to the MS prevalence rising to near that of Shanghai [25]. Worldwide, diverse races and diet cultures might be associated with MS prevalence. Based on the IDF definition, MS prevalence were reported to be 31.4% in India, 27.5% in Malaysia, and 32.8% in Mongolia, which were higher than the corresponding values in China and Jiangxi province [18, 23, 27].

More participants were inclined to be diagnosed with MS by the IDF criteria. Both diagnostic criteria have different cutoffs for BP, TC and glucose levels. Besides, IDF criteria rely on WC to define central obesity as a preliminary condition for diagnosing MS, while CDS criteria are based on BMI. This discrepancy might also lead to the opposite outcome of prevalence observed between genders. In this study, we found that about 20% women had low BMI (< 25 kg/m^2^) with over 80 cm WC, while 8% men had low BMI (< 25 kg/m^2^) with over 90 cm WC. On the other hand, over 23% men had higher BMI (> 25 kg/m^2^) with smaller WC (< 90 cm), and 7% women had higher BMI (> 25 kg/m^2^) with smaller WC (< 80 cm). In other words, females had higher WC with lower BMI, while males had higher BMI with lower WC. Similar results were reported in the Chinese population between 2010 and 2013, but not in Gu’s study performed in 2005 [8, 15, 26]. This suggests that sex-specific criteria need to be considered in diagnosing MS.

The associated factors for MS were evaluated by multiple logistic regression analysis in this study. Low education and the menopausal state were independent associated factors for adults in Jiangxi province. As shown above, individuals with low education had a higher risk of developing MS diagnosed by CDS criteria. We assumed that urban residents had higher odds of getting education resources, and received more information about balanced diet. As a tradition, the diet habit in Jiangxi province is fatty and salty. Blood TG levels, closely related to diet, were higher in rural individuals, suggesting the traditional diet habit is more common in rural areas of Jiangxi province. Therefore, a higher prevalence of MS in rural areas was observed in this study, regardless of the diagnostic criteria. Hence, a healthy diet should be recommended in rural areas of Jiangxi province. Similar to a previous study, a significant negative association of non-menopausal state with MS prevalence was shown in women [28]. This association is likely mediated by the testosterone/estradiol ratio. However, when compared with other regions in the world, region-specific factors may exist, such as alcohol consumption had a major association with MS in Mongolian population [18]. Therefore, region-specific measurement was needed to prevent the increase of MS prevalence.

The definition of MS remains controversial. It was reported that IDF and ATP III criteria have good consistency [24]. Here, we compared two other systems, including IDF and CDS criteria. It is well-known that China is a large population country, with the geography, climate, living conditions and diet varying from south to north. Identical MS criteria may not be suitable for different people. To the best of our knowledge, IDF criteria are more suitable to northwest Chinese individuals who have elevated WC compared with the others [13]. Besides, different criteria have distinct predictive values in prognosis. The IDF criteria have a reduced value for predicting all-cause mortality in T2DM patients, though it is better than ATP-III criteria [29].

This study had several strengths. Firstly, rigorous training was performed for all study staff before survey initiation. Secondly, detailed information about medications for metabolic disorders were collected. In several previous national or regional studies reporting high MS prevalence in the Chinese population, medications for lipid-, glucose- or blood pressure-lowering were not documented [14]. Therefore, the actual prevalence of MS could have been underestimated. As an epidemiological survey, this study also had limitations. Since only one urban community and four rural villages were included, the sample was small and only partially representative. Besides, our study did not investigate physical activity and dietary habit, which may miss some important information. Furthermore, the occurrence of negative outcome correlated with MS was not predicted, and further investigation is required for clarification.

## Conclusions

To the best of our knowledge, this was the latest study on MS prevalence in Jiangxi province. In conclusion, MS prevalence is high in Jiangxi province. Considering the unhealthy lifestyle, education is urgently needed to prevent the rapid increase of MS prevalence.

## Supplementary information

**Additional file 1: Table S1.** Criteria of metabolic syndrome diagnosis in the IDF and CDS systems.

## Data Availability

The dataset of the current study is available from the corresponding author upon reasonable request.

## Abbreviations

BMI: Body Mass Index
BP: Blood Pressure
CDS: Chinese Diabetes Society
CVD: Cardiovascular Disease
HbA1c: Glycosylated Hemoglobin A1c
HR: Heart Rate
IDF: International Diabetes Federation
MS: Metabolic Syndrome
SE: Standard Error
T2DM: Type 2 Diabetes
UA: Uric Acid
WC: Waist Circumstance
