# Supplementary material for: Prevalence and associated factors of metabolic syndrome in adults: a population-based epidemiological survey in Jiangxi province, China
Source: BMC Public Health. 2020 Jan 30;20:133. doi: 10.1186/s12889-020-8207-x (PMC6993347; doi:10.1186/s12889-020-8207-x)
Supplement: Supplementary file 1 — Additional file 1: Table S1. Criteria of metabolic syndrome diagnosis in the IDF and CDS systems. [file 12889_2020_8207_MOESM1_ESM.docx]

**Supplementary Table 1.** Criteria of metabolic syndrome diagnosis in the IDF and CDS systems

| MS components | IDF criteria |  | CDS criteria |
| --- | --- | --- | --- |
| To be diagnosed as MS | Central obesity plus any two other components |  | Any three or all of the following components |
| Obesity | Waist circumference |  | Body mass index |
| Men | ≥ 90 cm for Chinese men |  | ≥ 25 kg/m²  ≥ 25 kg/m² |
| Women | ≥ 80 cm for Chinese women |  |  |
| Metabolism disorder of lipids |  |  | Any of the following disorders |
| Serum triglyceride level | ≥ 1.7 mmol/L or specific treatment for dyslipidemia |  | ≥ 1.7 mmol/L |
| HDL cholesterol |  |  |  |
| Men | < 1.03 mmol/L or specific treatment for dyslipidemia |  | < 0.9 mmol/L |
| Women | < 1.29 mmol/L or specific treatment for dyslipidemia |  | < 1.0 mmol/L |
| Blood pressure | SBP ≥ 130 mmHg or DBP ≥ 85 mmHg or specific treatment for previously diagnosed hypertension |  | SBP≥140 mmHg or DBP ≥ 90 mmHg or specific treatment for previously diagnosed hypertension |
| Serum glucose level | Fasting blood glucose ≥ 5.6 mmol/L or specific treatment for previously diagnosed T2DM |  | Fasting blood glucose ≥ 6.1 mmol/L or OGTT-2h BG ≥ 7.8 mmol/L or specific treatment for previously diagnosed T2DM |

MS, metabolic syndrome; IDF, International Diabetes Federation criteria; CDS, Chinese Diabetes Society criteria; T2DM, type 2 diabetes mellitus; HDL cholesterol, high density lipoprotein cholesterol; SBP, systolic blood pressure; DBP, diastolic blood pressure; OGTT-2h BG, blood glucose of 2 hours oral glucose tolerance test.
